# Study on the Equivalence Transformation between Blasting Vibration Velocity and Acceleration

**DOI:** 10.3390/s24061727

**Published:** 2024-03-07

**Authors:** Chong Yu, Jiajun Wu, Haibo Li, Yongan Ma, Changjian Wang

**Affiliations:** 1 State Key Laboratory of Geomechanics and Geotechnical Engineering, Institute of Rock and Soil Mechanics, Chinese Academy of Sciences, Wuhan 430071, China; wujiajun21@mails.ucas.ac.cn (J.W.); hbli@whrsm.ac.cn (H.L.); mayongan22@mails.ucas.ac.cn (Y.M.); wangchangjian23@mails.ucas.ac.cn (C.W.); 2 University of Chinese Academy of Sciences, Beijing 100049, China

**Keywords:** seismometer, blasting vibration, noise reduction, wavelet packet, attenuation law

## Abstract

The evaluation of blasting vibrations primarily hinges on two physical quantities: velocity and acceleration. A significant challenge arises when attempting to reference the two types of vibration data in relation to one another, such as different types of seismometers, noise, etc., necessitating a method for their equivalent transformation. To address this, a transformation method is discussed in detail with a case study, and equations for the ratio (Ra) of the particle peak velocity (PPV) to the particle peak acceleration (PPA) are proposed. The findings are twofold: (1) The conventional data conversion processes often suffer from low accuracy due to the presence of trend terms and noise in the signal. To mitigate this, the built-in MATLAB function is used for trend term elimination, complemented by a combined approach that integrates CEEMDAN with WD/WDP for noise reduction. These significantly enhance the accuracy of the transformation. (2) This analysis reveals a positive power function correlation between Ra and the propagation distance of the blast vibrations, contrasted by a negative correlation with the maximum charge per delay. Intriguingly, the Ra values observed in pre-splitting blasting operations are consistently lower than those in bench blasting. The established Ra equations offer a rapid, direct method for assessing the transformation between the PPV and PPA, providing valuable insights for the optimization of blasting design.

## 1. Introduction

Blasting inevitably generates blasting vibration, which must be considered in blasting design. However, controlling the physical quantities for blast vibration in various buildings and structures is different, e.g., velocity is used for houses, buildings, tunnels and concrete etc., whereas acceleration is adopted for nuclear power plants and precision instruments affected by seismic inertial force [1,2,3,4]. Moreover, vibration acceleration is often chosen as the dynamic load during structural dynamic response calculations [5,6]. Due to phased construction and design modifications, there may be instances where only velocity or acceleration is initially monitored, but subsequently, another physical quantity becomes necessary for computation or blast design. To maximize the data utilization, the equivalent transformation of velocity and acceleration emerges as a practical problem requiring resolution.

(1) At present, there are three types of vibration sensors: displacement sensors, velocity sensors, and acceleration sensors. Each of these sensors is designed to monitor specific aspects of vibration. The displacement sensor measures the amplitude of the vibration, the velocity sensor quantifies the energy, and the acceleration sensor gauges the impact force. In the low-frequency range, the intensity of vibration is proportional to displacement; in the mid-frequency range, it is proportional to velocity; and in the high-frequency range, it is proportional to acceleration. In the field of blasting vibration, inductive-type transducers are commonly used to measure velocity. These transducers utilize the magnetoelectric (ME) effect to convert motion velocity into an induced potential output in the coil. These sensors operate without the need for an external power source, instead directly harnessing the mechanical energy of the object being measured and converting it into an electrical signal output [7,8]. Because ME enables the high-sensitivity transformation from a magnetic signal into an electric signal, researchers have found it an attractive option for developing magnetic field sensors and current sensors [9]. For acceleration measurements, piezoelectric acceleration sensors are commonly used. These sensors are a type of inertial sensor that utilizes the piezoelectric effect of quartz crystals, such as lead zirconate titanate [10,11,12].

(2) The similarities and differences between two signals in phase, amplitude, and frequency [13], for a sinusoidal velocity signal, are denoted as:

(1) v(t)=Asin(ωt+φ)

where *A* represents the maximum amplitude, φ is the initial phase, *ω* = 2*πf* is the angular frequency, and *f* denotes the frequency. Differentiating the velocity signal yields:

(2) a(t)=ωAsin(ωt+φ+π/2)

This reveals that for the sinusoidal signal, the angular frequency and the frequency–response function of velocity and acceleration are identical, albeit with a 90° phase difference. Furthermore, a linear relationship exists between the acceleration amplitude (*a_max_ = Aω*) and the velocity amplitude (*v_max_ = A*):

(3) $$a_{max}=2\pi fv_{max}$$

(3) Transforming acceleration signals into velocity signals. This can be achieved using time-domain integration [14] or frequency-domain integration [15].

Time-domain integration directly integrates an acceleration signal into a velocity signal, typically employing the trapezoidal formula, Simpson formula, or Newton–Cotes formula [16,17]. Frequency-domain integration first transforms a time-domain acceleration signal into a frequency-domain signal using Fourier transform (FT), then integrates in the frequency domain, and, finally, reverts the frequency-domain signal back into the time-domain signal using Inverse Fourier Transform (IFT). While frequency-domain integration effectively circumvents the accumulation and amplification of minor errors in time-domain integration, it is sensitive to a lower cut-off frequency, and inappropriate parameter selection can result in significant integration errors. Given that measured blasting vibration signals often contain uncertain random noise and multiple frequency components, accurately and quantitatively calculating the cut-off frequency using the frequency-domain integration method is challenging. Therefore, time-domain integration is more suitable for transforming the blasting vibration acceleration into velocity.

This study conducts field blasting tests to discuss the transformation between vibration velocity and acceleration and to analyze the ratio of these two physical quantities and the differences in their vibration attenuation laws. The organization of this paper is as follows. In Section 2, field blasting tests are carried out, the performance of the seismometer is analyzed, and monitoring data on the field velocity and acceleration are obtained. In Section 3, in view of the transient, nonlinear, and wide-frequency characteristics of blasting vibration signals, a comprehensive method based on the time domain and frequency domain to remove trend items and denoise is proposed. In Section 4, the ratio of the velocity amplitude to the acceleration amplitude is analyzed, and the ratios of the two blasting methods (pre-splitting blasting and bench blasting) are also compared. In Section 5, the attenuation laws of the blasting vibration velocity and acceleration are analyzed.

## 2. Field Blasting Tests

### 2.1. Descriptions of the Blast Tests

The blasting test site is situated at Haiyang nuclear power plant (HNPP) in Shandong Province, China, characterized by a lithology primarily composed of fine sandstone and siltstone. The blasting tests employed No. 2 rock emulsion explosives, millisecond detonators, and high-energy capacitive initiators, incorporating two blasting methodologies: pre-splitting and bench blasting. The parameters for the blasting design are detailed in Table 1.

**Figure 1:**
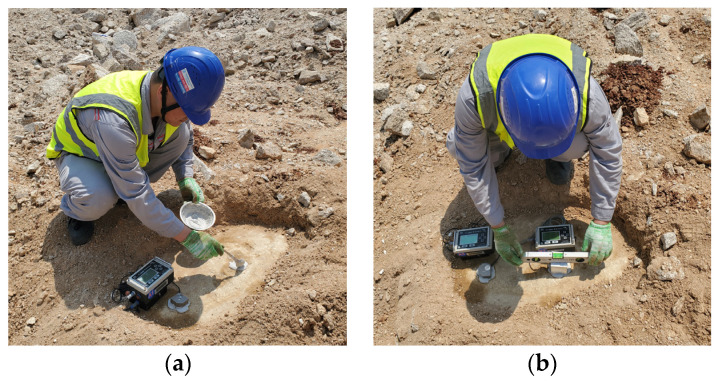
Layout of velocity and acceleration seismometers. (**a**) Gypsum bonding. (**b**) Leveling seismometers.

The nearest monitoring point is a mere 10 m from the blast hole, and the furthest is 598 m. The velocity/acceleration seismometers were bonded to the bedrock with gypsum and leveled by a level ruler. The orientations of the two seismometers were aligned parallel to each other. The layout of the seismometers is depicted in Figure 1. Sand and mud were selected as the stemming material, with the explicit exclusion of any flammable substances. Throughout the stemming process, it was imperative to protect the detonators within the borehole. The quality and length of the stemming were meticulously controlled to avert the formation of voids and looseness.

The blasting tests were conducted in two batches. The initial batch (5 blast tests) focused on the method of equivalent transformation between velocity and acceleration. The second batch (32 blast tests) was incorporated to analyze the attenuation laws of velocity and acceleration and the ratio of velocity amplitude to acceleration amplitude.

### 2.2. Seismometer Performance

A seismometer is an instrument that transduces mechanical signals into electrical ones. Its primary function is to execute an electrical simulation of vibrations with minimal distortion, thereby mirroring the dynamic attributes of vibrational waves. Various types of seismometers monitor different physical quantities, each with distinct data characteristics. To reliably and effectively compare and analyze different physical quantities (velocity and acceleration) of the same vibration, it is essential that the seismometers meet the frequency response, range, and accuracy requirements of the vibrational signal.

**Figure 2:**
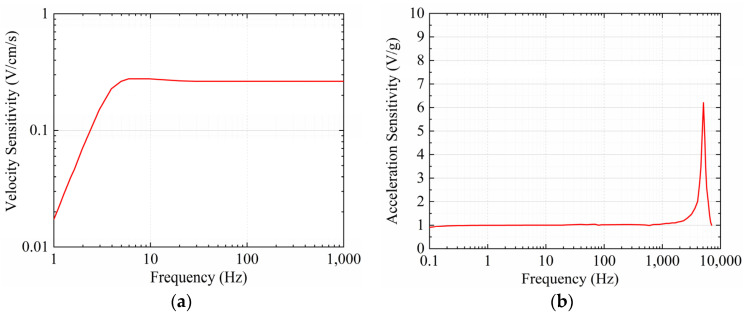
Frequency–response curves of seismometers. (**a**) TCS-B3 velocity seismometer. (**b**) LC0161D acceleration seismometer.

In this study, a TCS-B3 velocity seismometer and an LC0161D acceleration seismometer were employed for field tests. The TCS-B3 has a testing range of 35 cm/s and an accuracy of 0.001 cm/s. The LC0161D has an acceleration range of 5 g and an accuracy of 0.02 mg. The frequency response of a seismometer, a crucial performance index, reflects the sensitivity variation in the seismometer with frequency. Figure 2 illustrates the frequency–response curves of both seismometers. The linear segment in the curve represents the optimal monitoring range, and other segments may introduce errors. However, it should be noted that the frequency–response curve of the entire monitoring system not only depends on the seismometer but is also influenced by factors such as the seismometer packaging, the charge amplifier, and the bonding between the seismometer and the measured object. As the manufacturer’s recommended performance indices, the frequency–response range for effective velocity monitoring is 5–300 Hz, and for acceleration, it is 0.1–1000 Hz.

**Figure 3:**
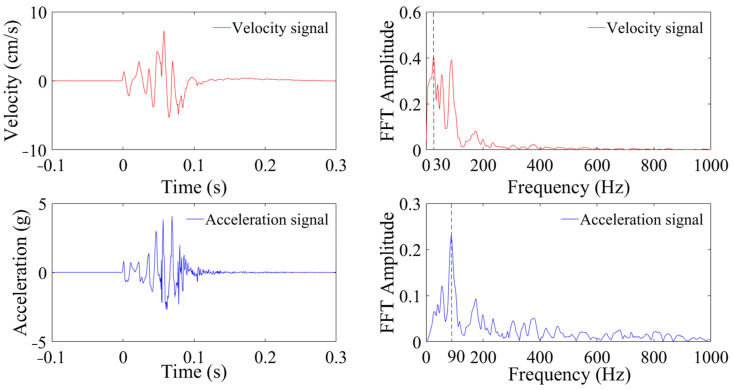
Vibration signal monitored at 10 m.

**Figure 4:**
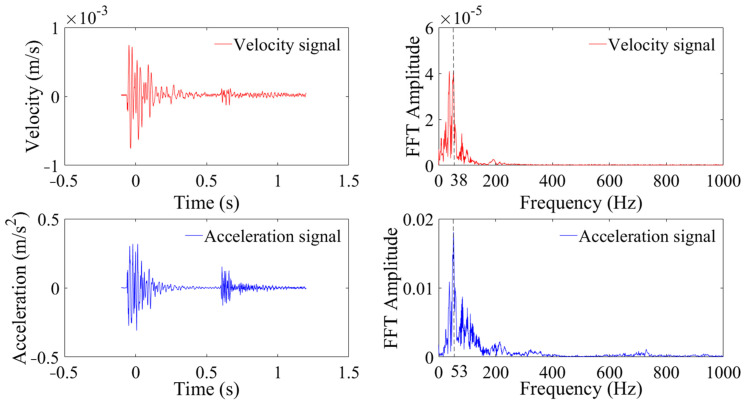
Vibration signal monitored at 235 m.

Figure 3 and Figure 4 show the measured velocity, acceleration, and FT analysis at distances of 10 m and 235 m from the blasting area during the first blasting test. As depicted in Figure 3, the particle peak velocity (PPV) at 10 m is 7.26 cm/s, with a peak frequency of 30 Hz and a frequency distribution primarily concentrated within 5–200 Hz. The peak acceleration is 4.25 g, with a peak frequency of 90 Hz and a frequency distribution primarily within 5–400 Hz. In Figure 4, the peak velocity at 235 m is 0.08 cm/s, with a peak frequency of 38 Hz and a frequency distribution primarily within 5–110 Hz. The peak acceleration is 32 mg, the peak frequency is 53 Hz, and the frequency domain is primarily within 20–245 Hz. The frequency distributions of both velocity and acceleration fall within the linear segment of the frequency–response curves, thereby satisfying the frequency–response requirements of the vibration signals. It is also observed that the velocity signals exhibit a multi-peaked frequency distribution, while accelerations are characterized by a single peak. Compared to the vibration velocity, the frequency distribution of the acceleration shifts towards the higher-frequency band.

## 3. Velocity and Acceleration Transformation

### 3.1. Removal of the Trend Term

Equations (1) and (2) reveal that, theoretically, the sinusoidal velocity and acceleration signals can be equivalently transformed using differentiation and integration. However, blast vibration signals contain multiple frequency components and nonlinear and non-stationary characteristics. The transformation effect is thus a question. Two sets of data at 10 m and 235 m from the explosion source were selected for analysis.

**Figure 5:**
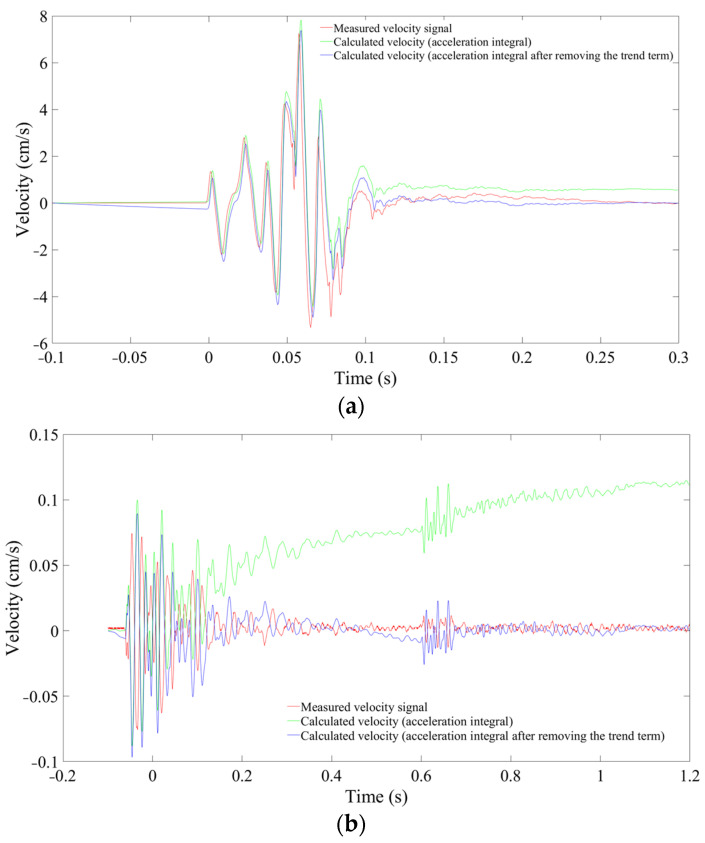
Measured and calculated velocity signals. (**a**) 10 m. (**b**) 235 m.

**Figure 6:**
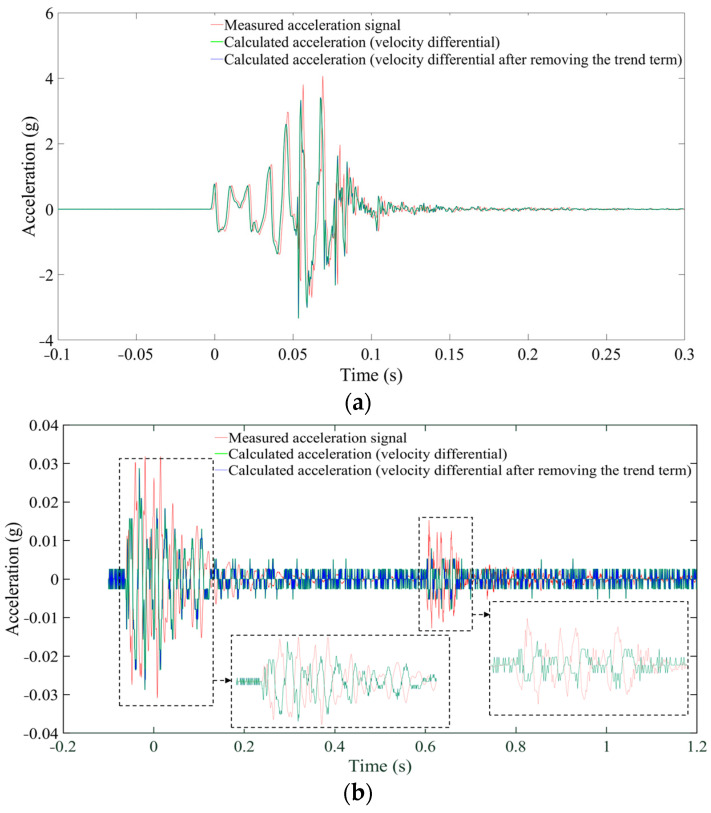
Measured and calculated acceleration signals. (**a**) 10 m. (**b**) 235 m.

The relative error ($E_{va}$) of the absolute peak value is employed as the evaluation index of the calculation error. Its definition formula is as follows, and the calculation results are listed in Table 2.

(4) $$E_{va}=\frac{max\left\{abs\left[va{\left(t\right)}^{\prime }\right]\right\}-max\left\{abs\left[va(t)\right]\right\}}{max\left\{abs\left[va(t)\right]\right\}}\times 100\%$$

where $va{\left(t\right)}^{\prime }$ is the calculated velocity or acceleration, and va(t) is the measured valve. In Figure 5, the velocity obtained by integrating the measured acceleration exhibits significant uncertainty, $E_{va}$ = 7.75% at a distance of 10 m and $E_{va}$ = 52.02% at 235 m. This is due to the amplification of the baseline drift in the measured acceleration time–history curves during integration. Consequently, baseline correction is essential to eliminate the trend term prior to integration. The ‘detrend’ function in MATLAB is used to remove the best straight-fit line from the data points, returning a signal with a mean value of 0. Following this operation, the error is significantly reduced but still reaches up to 28.13% at 235 m. Overall, the effect of the calculated acceleration (Figure 6) is superior to that of the calculated velocity, but there are still certain discrepancies compared to the measured data.

### 3.2. Noise Reduction

**Figure 7:**
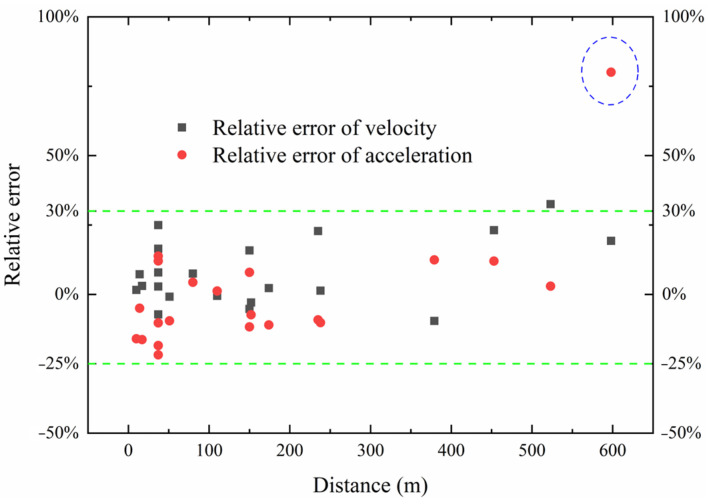
Relative error between measured and calculated amplitudes in the first batch blasting tests.

**Figure 8:**
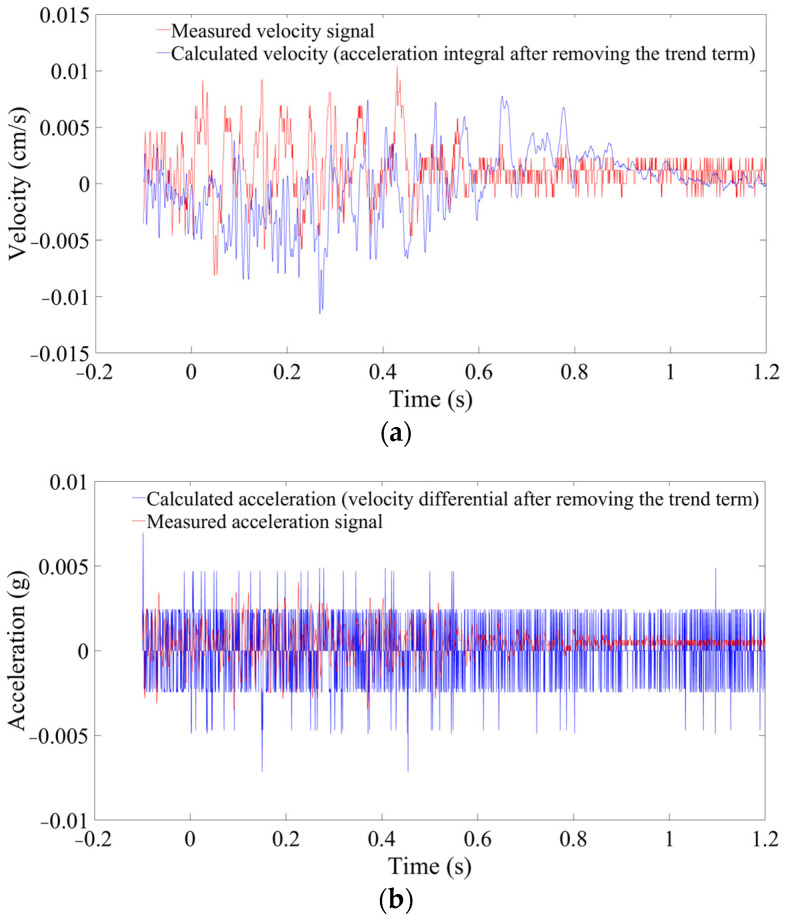
Transformation between velocity and acceleration (monitored at 598 m). (**a**) Velocity. (**b**) Acceleration.

In the first phase of blasting tests, the relative error of the calculated velocity after the removal of the trend term is shown in Figure 7, which indicates that the relative error is primarily concentrated between −25 and 30%. However, the error increases abnormally at longer propagation distances, e.g., at 598 m, it escalates to over 80%, and the transformation results are shown in Figure 8. Due to the long propagation distance, the amplitude of the blast vibration signal diminishes, and the signal-to-noise ratio (SNR) is low (SNR = 6.28), resulting in unsatisfactory transformation results and a poor match between the calculated and measured curves. Therefore, blast vibration signals with a small SNR should be denoised prior to calculation.

Currently, noise reduction methods in various fields such as fault diagnosis [18], seismic exploration [19], medical images [20], military [21], and others can provide references for noise reduction for blasting vibration signals. Common noise reduction methods include FT, wavelet decomposition (WD), wavelet packet decomposition (WPD), and Hilbert–Huang Transform (HHT). FT only handles the noise of the stationary signal and fails to reflect the transient characteristics of the blasting vibration signal. WD is a time-frequency analysis tool that decomposes a signal into wavelet coefficients of different frequency bands, then selects an appropriate threshold to eliminate some of the wavelet coefficients, and finally reconstructs the retained wavelet coefficients to complete noise reduction for the signal. As an improved algorithm of WD, WPD continues to divide the high-frequency components that are not subdivided in the process of WD, which has a stronger ability to separate noise from signals. Although WD and WPD can be used for noise reduction analysis of non-stationary and non-linear signals, both WD and WPD are limited by the choice of wavelet basis function and decomposition level. The noise reduction effects are uncertain. HHT consists of two parts, empirical mode decomposition (EMD) and Hilbert transform, which can decompose the original signal into several intrinsic mode function (IMF) components without specifying wavelet basic functions and decomposition layers. It is an adaptive time-frequency analysis method, but EMD has the mode-mixing phenomenon. To solve this problem, complete ensemble empirical mode decomposition with adaptive noise (CEEMDAN) is proposed to solve the mode-mixing phenomenon. However, the common HHT noise reduction method is to directly remove the IMF component containing the noise, resulting in the loss of part of the effective signal and causing signal distortion.

In view of the transient, nonlinear, and wide-frequency-range characteristics of blast vibration signals, this study adopts the method of combining CEEMDAN and WD/WDP to reduce noise. Firstly, the noise-containing IMFs obtained using CEEMDAN are sieved based on correlation coefficients. Then, WD and WDP noise reduction are applied to these IMFs, and the effective information on these IMFs is preserved. Finally, the noise-reduced IMFs, together with noiseless IMFs, are reconstructed to obtain the final blast vibration signal.

The specific algorithmic steps of CEEMDAN are as follows [22]:

(1) Constructing the new signal *va_m_*(*t*)′ by adding white noise to the original signal *va*(*t*),

(5) $$va_{m}{\left(t\right)}^{\prime }=va\left(t\right)+\xi i\omega_{m}\left(t\right)$$

where *ξi* (*i* = 1, 2, …, *d*) is the noise standard deviation, and *d* is the number of IMFs generated using the CEEMDAN algorithm. *ω_m_*(*t*) (*m* = 1, 2, …, *M*) is the white noise added at the *m*-th time with a standard normal distribution, and *M* is the ensemble size, i.e., the number of added white noise terms.

(2) Defining the operator *E_k_*(•) as the *k*-th order modal component generated using the EMD algorithm and taking the mean value of the first modal component of *va_m_*(*t*)′ as the first order of *va*(*t*).

(6) $${\mathrm{IMF}}_{1}(t)=\frac{1}{M}\sum_{m=1}^{M}E_{1}(va_{m}(t))$$

The first residue is

(7) $$RS_{1}(t)=va(t)-{\mathrm{IMF}}_{1}(t)$$

(3) Continuing to introduce the first-order component of the EMD of the white noise signal and combining it with the first residue to eliminate the error caused by the noise in the original signal, then the second-order is

(8) $${\mathrm{IMF}}_{2}(t)=\frac{1}{M}\sum_{m=1}^{M}E_{1}(RS_{1}(t)+\xi_{2}E_{1}(\omega_{m}(t)))$$

The second residue is

(9) $$RS_{2}(t)=RS_{1}(t)-{\mathrm{IMF}}_{2}(t)$$

(4) Similarly, to step (3), the (*i* + 1)-th modal component is

(10) $${\mathrm{IMF}}_{i+1}(t)=\frac{1}{M}\sum_{m=1}^{M}E_{1}(RS_{i}(t)+\xi_{i+1}E_{i}(\omega_{m}(t)))$$

(5) Until the obtained residue is no longer able to be decomposed (the residue is monotone function). The final residue is

(11) $$RS_{d}(t)=va(t)-\sum_{i=1}^{d}{\mathrm{IMF}}_{i}(t)$$

Then, the original signal can be expressed as

(12) $$va(t)=\sum_{i=1}^{n}{\mathrm{IMF}}_{i}(t)+RS_{d}(t)$$

**Figure 9:**
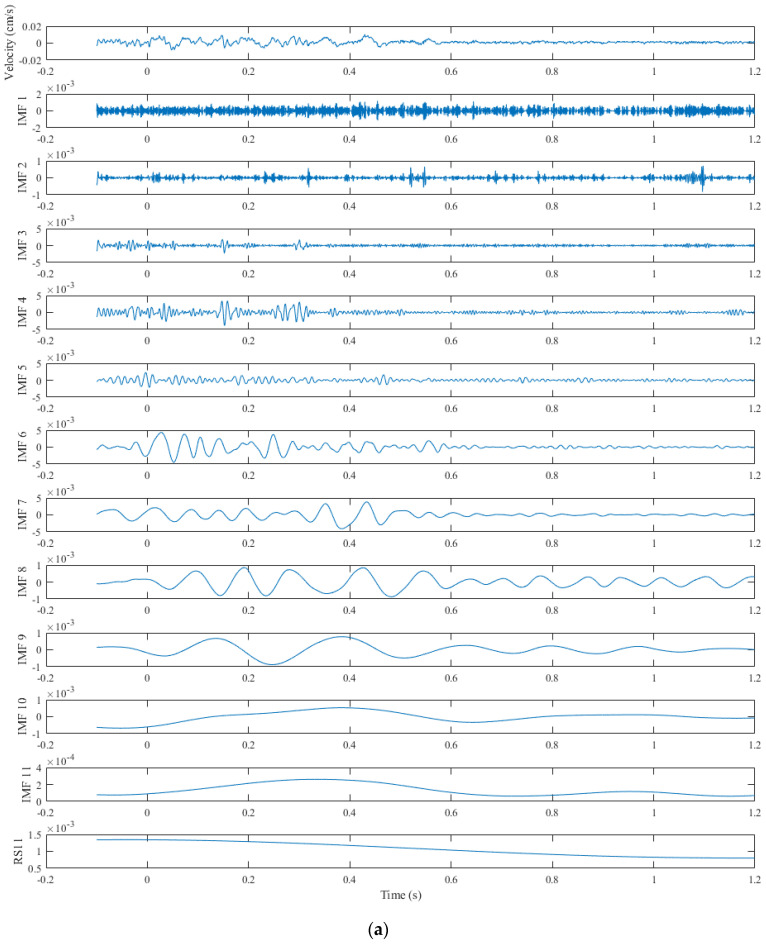

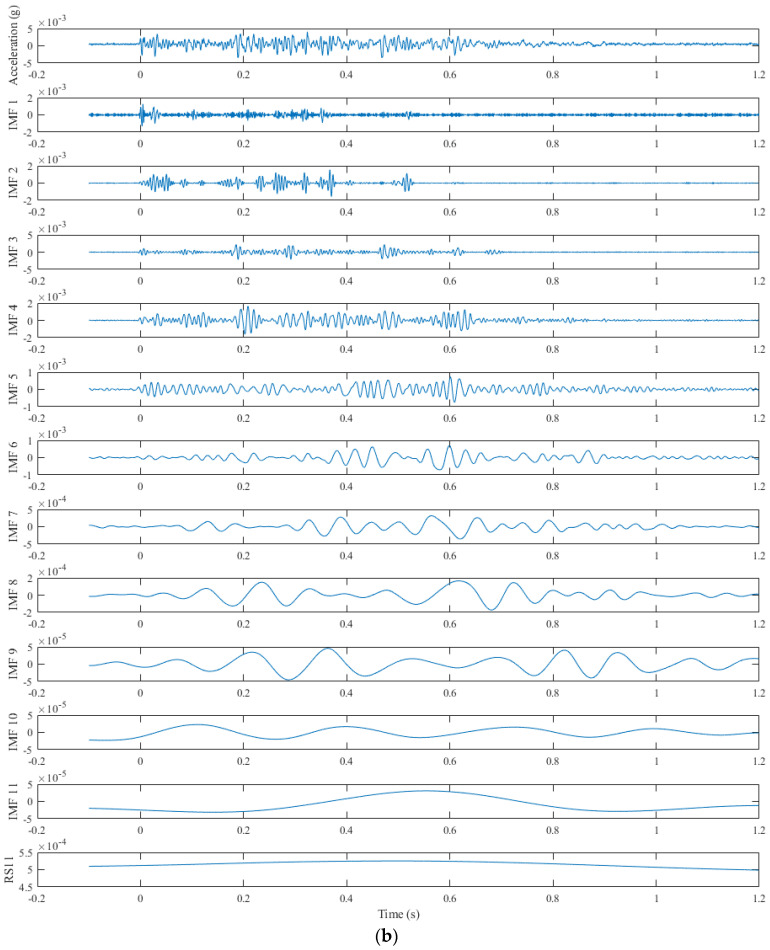
Mode decomposition results based on CEEMDAN (monitored at 598 m). (**a**) Velocity signal. (**b**) Acceleration signal.

According to the above steps, the IMFs of the blast signals are shown in Figure 9. As can be seen, CEEMDAN extracts all the IMFs in the signal itself, from high-frequency to low-frequency, and the residual term *RS*_11_ is the trend term of the signal.

**Figure 10:**
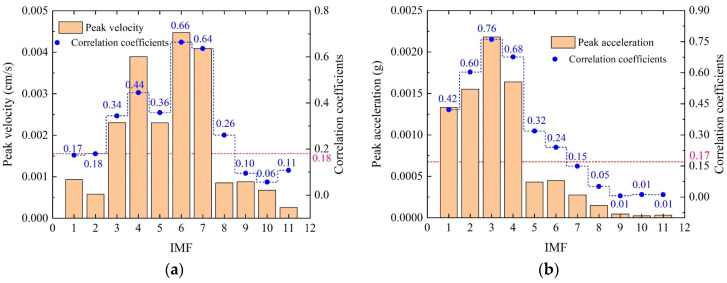
Correlation coefficients and peak value of IMFs. (**a**) Velocity signal. (**b**) Acceleration signal.

The equation of the correlation coefficient between the IMF and the original signal is

(13) $$r_{i}=\frac{Cov({\mathrm{IMF}}_{i},va(t))}{\sqrt{SD({\mathrm{IMF}}_{i})}\sqrt{SD(va(t))}}$$

where *r_i_* is the correlation coefficient of the *i*-th IMF component with the original signal *va*(*t*), *Cov*(IMF*_i_*, *va*(*t*)) is the covariance of the IMF*_i_* component and *va*(*t*), $\sqrt{SD({\mathrm{IMF}}_{i})}$ is the standard deviation of the IMF*_i_* component, and $\sqrt{SD(va(t))}$ is the standard deviation of *va*(*t*). The calculation results and the peak of the IMF are presented in Figure 10.

Ayenu-Prah and Attoh-Okine [23] established a threshold (*r_TH_*) to eliminate interference IMFs (not larger than this threshold) and retain predominant IMFs (larger than this threshold). Its expression is

(14) $$r_{TH}=\frac{max(r_{i})}{10\times max(r_{i})-3}$$

Considering that interfering IMFs still contain some valid information, this study used the threshold to determine noisy IMFs for the following noise reduction instead of directly removing the IMFs. The threshold values for the velocity and acceleration are calculated using Equation (14) as 0.18 and 0.17, respectively. Thus, IMF3–IMF8 of the velocity signal are the dominant IMFs, while IMF1, IMF2, and IMF9–IMF11 are noisy IMFs. IMF2–IMF6 of the acceleration signal are the dominant IMFs, while IMF7–IMF11 are noisy IMFs. It should be noted that IMF1 contains high-frequency characteristics far exceeding the blasting vibration frequency. Therefore, IMF1 is also considered to be a noisy IMF. All noisy IMFs are reconstructed for WD/WDP denoising analysis.

The main stages of the WD/WDP denoising analyses can be summarized as follows: decomposition, threshold quantization, and reconstruction. Among these stages, the choice of wavelet basis function, the method of threshold estimation, and the level of decomposition exert a certain influence on the noise reduction. The MATLAB wavelet analysis toolkit offers a variety of wavelet basis functions, including a Morlet wavelet, Daubechies wavelet (dbN), Symlets wavelet (symN), etc.

The empirical results have demonstrated that the sym7, sym8 [24], db6 [25], and db8 [26] wavelet basic functions are particularly effective in handling blast vibration signals. The ‘ddencmp’ function in MATLAB provides default values for denoising or compression for the critically sampled discrete WD or WPD. The default threshold estimation methods encompass ‘rigrsure’, ‘heursure’, ‘sqtwolog’, ‘minimaxi’.

The sampling frequency is set at 2000 Hz. In accordance with the Shannon sampling theorem, the Nyquist frequency is 1000 Hz. When the decomposition level reaches 8, the original signal will be decomposed into 2^8^ = 256 sub-bands in the frequency domain, with a minimum frequency band of 0–3.9 Hz.

Two indicators, the root mean square error (RMSE) and SNR, are often used to evaluate noise reduction. The expression of the RMSE and SNR are given in Equations (15) and (16), respectively,

(15) $$\mathrm{RMSE}=\sqrt{\sum_{n=1}^{N}{\left(\hat{f}(n)-f(n)\right)}^{2}/N}$$

(16) $$\mathrm{SNR}=10\log\left[\sum_{n=1}^{N}f^{2}(n)/\sum_{n=1}^{N}{\left(f(n)-\hat{f}(n)\right)}^{2}\right]$$

where $\hat{f}(n)$ is the signal after noise reduction, and f(n) represents the original signal. A smaller RMSE implies a closer resemblance of the denoised signal to the original signal. A higher SNR indicates a greater ratio of signal power to noise power.

In addition to the above two indicators, the smoothness of the curve (SM) is also utilized to evaluate noise reduction, which indicates the smoothness of the curve. The calculation formula of the SM [27] at a point *x_0_* is given in Equation (17),

(17) $${\mathrm{SM}|}_{x=x_{0}}=f(x_{0}+2h)-f(x_{0}-2h)-2\left[f(x_{0}+h)-f(x_{0}-h)\right]$$

where *h* is the sampling interval. ${\mathrm{SM}|}_{x=x_{0}}$ being closer to zero implies that the neighborhood of point *x*_0_ of the curve is smoother. The sum of the SM of all points on the denoising curve is denoted as SSM.

**Figure 11:**
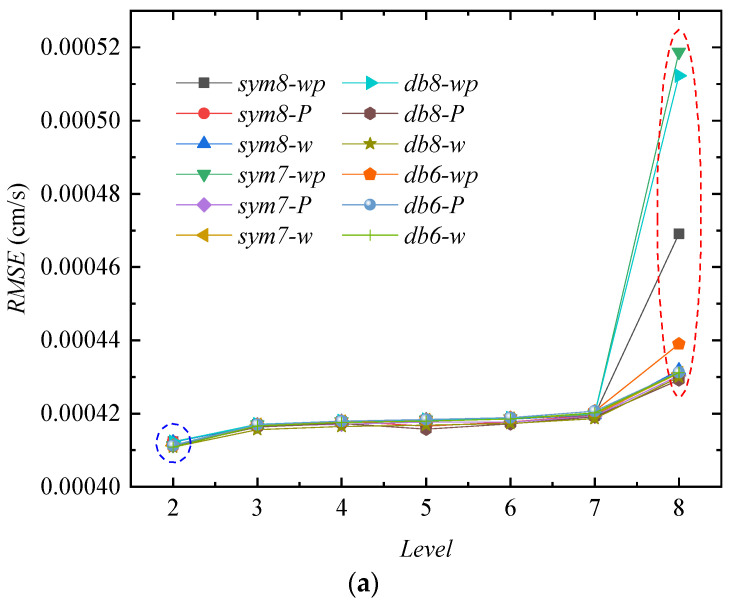

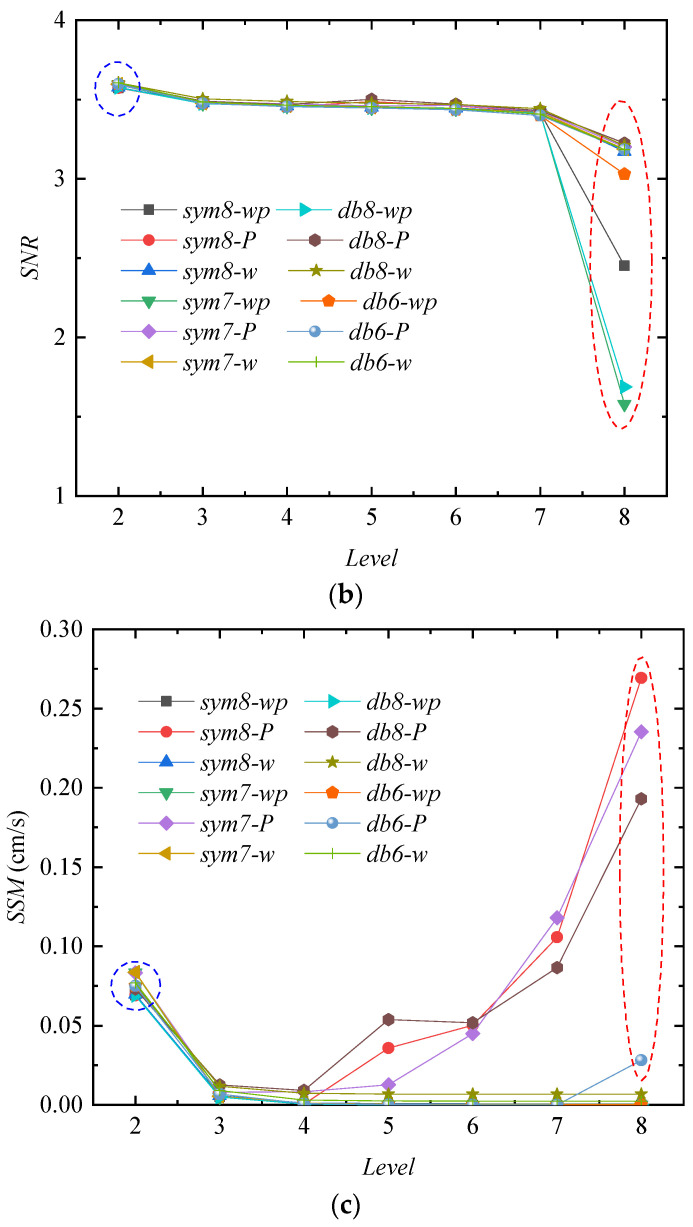
Noise reduction indicators for the velocity signal. (**a**) Variation in RMSE with decomposition level. (**b**) Variation in SNR with decomposition level. (**c**) Variation in SSM with decomposition level.

**Figure 12:**
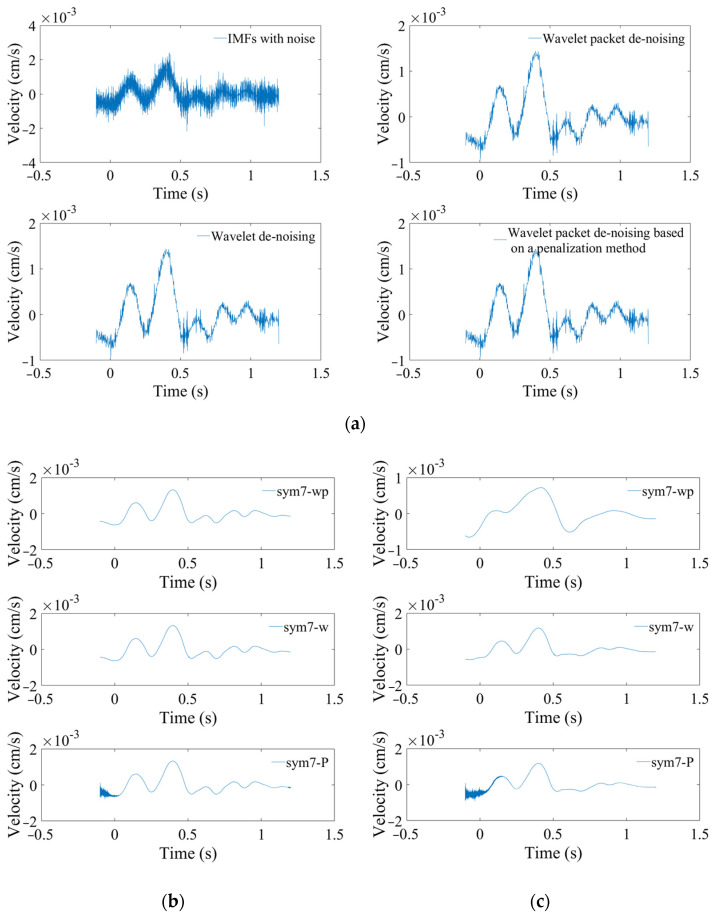
Noise reduction effects of the velocity signal. (**a**) At level 2 with *sym7*. (**b**) At level 7 with sym7. (**c**) At level 8 with *sym7*.

**Figure 13:**
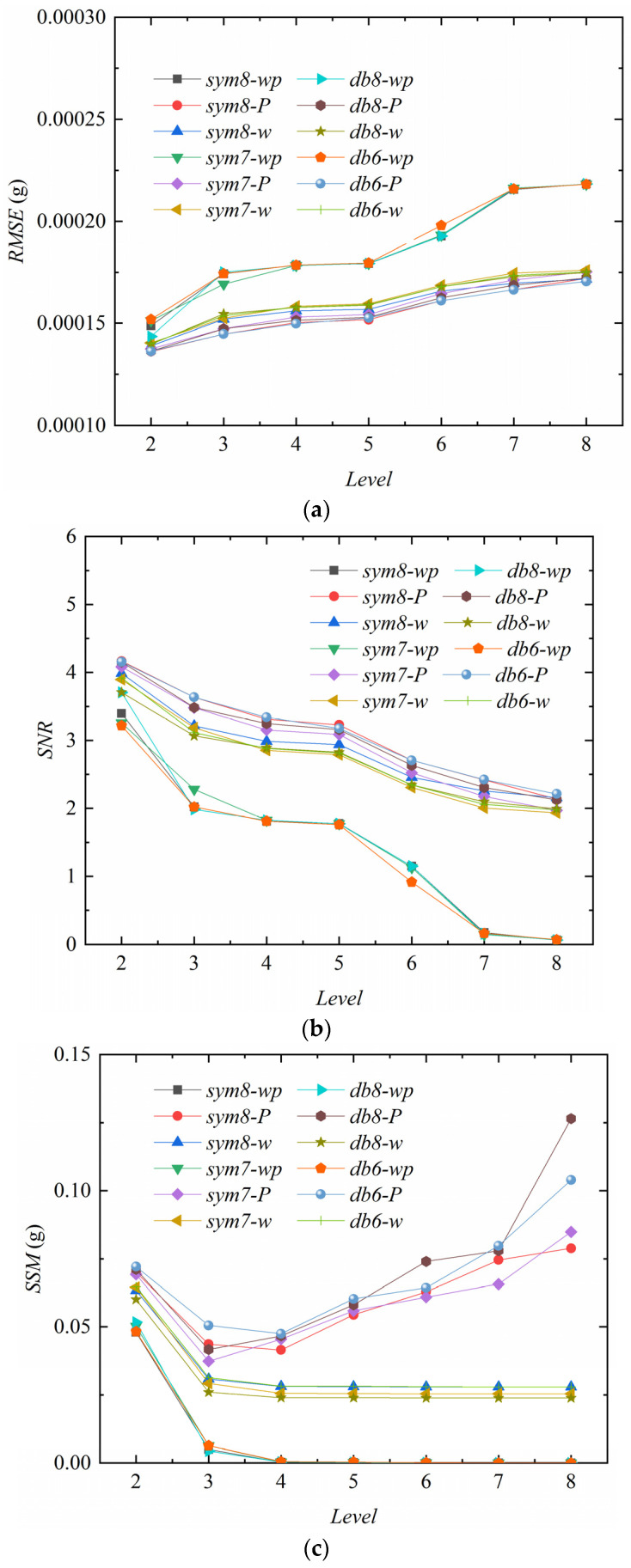
Noise reduction indicators for the acceleration signal. (**a**) Variation in RMSE with decomposition level. (**b**) Variation in SNR with decomposition level. (**c**) Variation in SSM with decomposition level.

**Figure 14:**
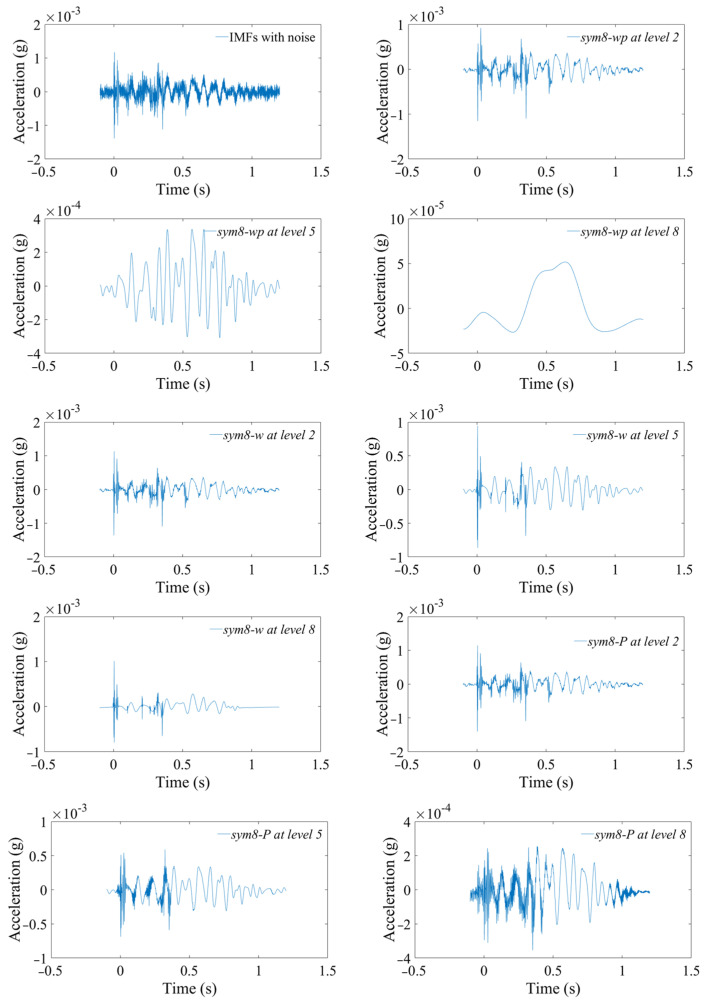
Noise reduction effects of the acceleration signal.

Figure 11, Figure 12, Figure 13 and Figure 14 compare the three indicators and noise reduction effects. In these figures, the following *w* represents a wavelet with the default threshold, *wp* represents a wavelet packet with the default threshold, and *P* represents a wavelet packet with a penalized threshold.

Figure 11a demonstrates that the RMREs of the various noise reduction methods are the smallest when the decomposition level is 2. The RMRE values slowly increase with the levels from 3 to 7, but when the level reaches 8, the RMREs of all the noise reduction methods increase rapidly, especially the *sym7-wp* method, which has a maximum increase of about 24%. Figure 11b reveals that the SNRs decrease slightly at level 3 compared to level 2 and then decrease slowly. When the level is 8, the SNRs decrease rapidly, and the *sym7-wp* method shows the largest decrease of about 54%. Figure 11c suggests that the SSMs of the three methods (*sym8-P*, *sym7-P*, and *db8-P*) first decrease and then increase rapidly.

Figure 12a is the curve after noise reduction with *sym7* at decomposition level 2. The results of the *sym7-wp*, *sym7-w*, and *sym7-P* methods are approximately the same and have the problem of incomplete noise reduction, resulting in unsmooth curves. As the levels increase to 5–7, the denoised curves are gradually smooth. Figure 12b is the curve at level 7 with *sym7*, in which *sym7-wp* and *sym7-w* are smooth, but some noise is still not removed for *sym7-P*, which is consistent with the characteristics reflected by the SSM in Figure 11c. *sym7-P* has a gradually increasing SSM value after the fifth level. Figure 12c is the result at level 8 with sym7; the curves are smooth after noise reduction (except for *sym7-P*) but the RMSEs increase and the SNRs decrease due to excessive noise reduction.

It is generally believed that the smaller the RMSE, the higher the SNR, and the lower the SSM, the better the noise reduction. Combined with the results on the RMSE, SNR, and SSM, the WD and WDP (with the default thresholds at 5–7 decomposition levels) can achieve good noise reduction results for this velocity signal.

Figure 13a,b suggest that these indicators of WDP with the default threshold are significantly different from other methods. The RMREs are large, and the SNRs are small. As the decomposition levels increase, the positive correlation of the RMRE and the negative correlation of the SNR are also greater than other methods. In Figure 13c, the SSM has three variation characteristics as the levels increase: (1) the SSMs decrease rapidly and then trend toward 0 steadily, such as *sym7-wp*, *sym8-wp*, *db6-wp*, *db8-wp*; (2) Similar to (1), but it tends to be close to a non-zero value, such as *sym7-w*, *sym8-w*, *db6-w*, *db8-w*; (3) the SSMs first decrease and then increase rapidly, such as *sym7-P*, *sym8-P*, *db6-P*, *db8-P*.

Through the analysis of the RMRE, SNR, and SSM, the noise reduction for this acceleration signal is mainly determined by the decomposition level and the threshold estimation method, which is weakly influenced by these four wavelet basic functions. Take *sym8* as an example to analyze the noise reduction effect, as shown in Figure 14. As the decomposition levels increase, WPD with default threshold is prone to excessive noise reduction, removing effective signals, and causing waveform distortion. Relatively speaking, WD with the default threshold is stable, and the waveform does not change drastically. The penalization method is between the two.

**Figure 15:**
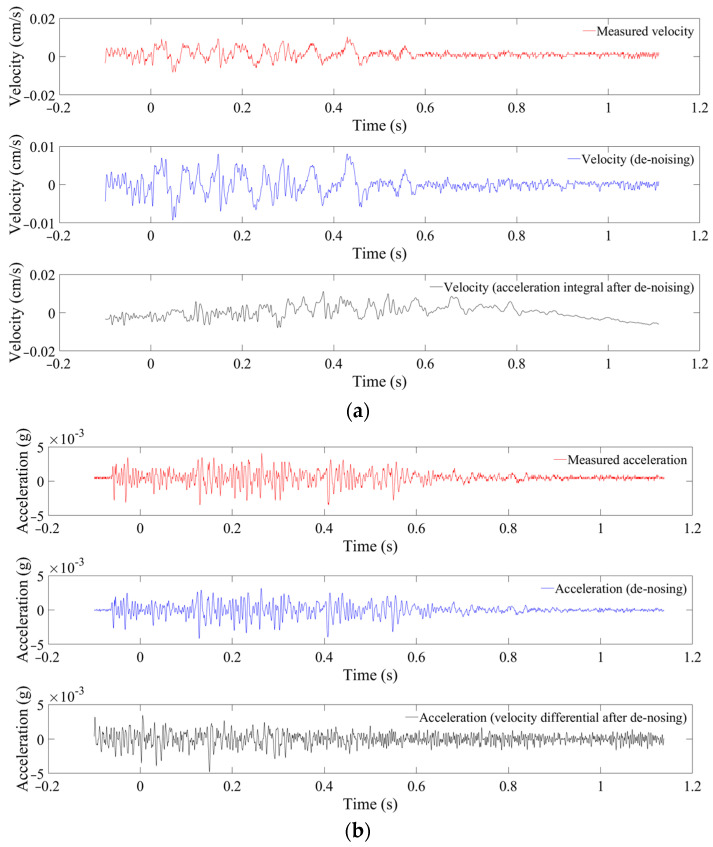
Transformation between velocity and acceleration after denoising (monitored at 598 m). (**a**) Velocity. (**b**) Acceleration.

Through the above analysis, WPD with the default threshold and sym7 at level 7 is selected for the velocity noise reduction. WD with the default threshold and sym8 at level 5 is selected for the acceleration noise reduction. After that, the denoised IMFs are reconstructed with the above noiseless IMFs. Finally, the denoised signals are obtained. Transformations of velocity and acceleration are carried out as shown in Figure 15. The relative error at 598 m is reduced to less than 18%. Therefore, when the SNR is small, the noise reduction method proposed in this paper can greatly reduce the error.

**Figure 16:**
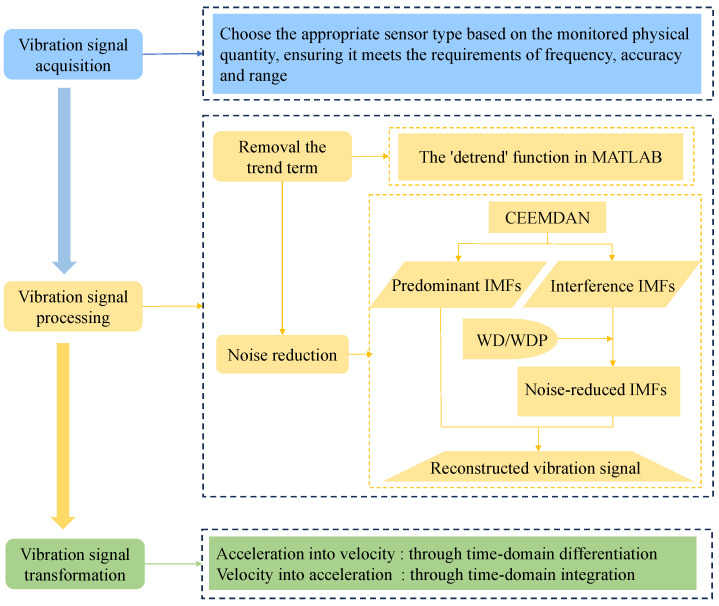
Flow chart of the conversion method.

Through the above analysis, the conversion method for blasting vibration velocity and acceleration is summarized; see Figure 16.

## 4. The Ratio of Velocity Amplitude to Acceleration Amplitude

For trigonometric signals, a 2*πf*-times relationship exists between the peak acceleration and the peak velocity. However, blast vibration signals are non-periodic, nonlinear, and non-stationary, raising the question of the proportionality between velocity and acceleration.

### 4.1. Influence of the Propagation Distance

**Figure 17:**
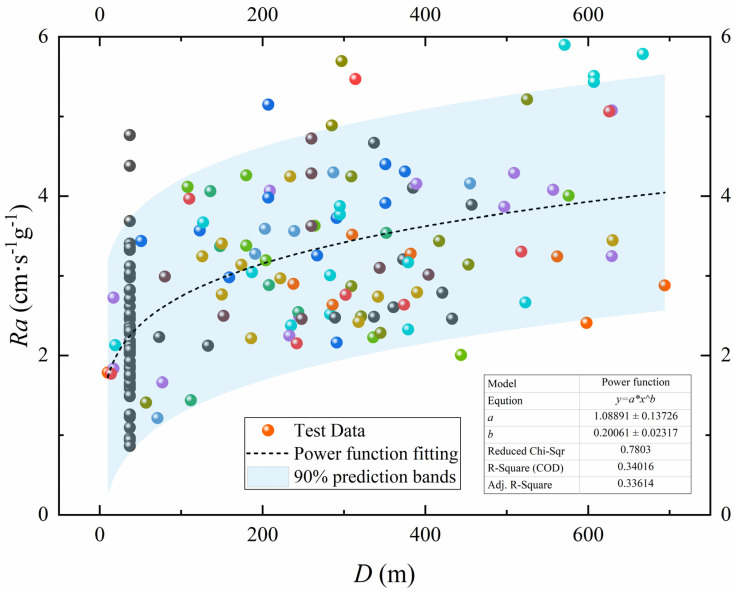
Relationship between *Ra* and *D*.

A total of 164 sets of data from two batches of tests are analyzed, and the ratio of the PPV to the PPA (*Ra =* PPV/PPA) is shown in Figure 17. The PPV is in cm/s, and the PPA is in g. *Ra* exhibits a power function relationship with the propagation distance (*D*, m) as follows:

(18) $$Ra=1.08891D^{0.20061}$$

as *D* increases, *Ra* gradually increases, but the rate of increase gradually decreases. This is primarily due to the fact that as the blast vibration propagates outward, the wavefront enlarges and the energy density decreases, coupled with the absorption of the vibration energy by the medium, eventually leading to a decrease in amplitude. The degree of decrease is related to the signal frequency, with higher frequencies experiencing faster attenuation. The frequency of the acceleration signal is greater than that of the velocity signal; therefore, the acceleration attenuation is greater than the velocity attenuation, resulting in a gradually increasing *Ra*.

Figure 17 presents the adjusted coefficient of determination (COD) of the fitting equation is only 0.33614, indicating that Ra is not only related to the propagation distance of the blasting vibration but also to the attenuation difference between velocity and acceleration. To improve the prediction accuracy, it is more reasonable to use prediction bands. At a 90% confidence level, the prediction bands are

(19) $$\mathrm{Upper}\;\mathrm{limit}\;\left(Rau\right),Rau=1.22617D^{0.22378}$$

(20) $$\mathrm{Lower}\;\mathrm{limit}\;\left(Ral\right),Ral=0.95165D^{0.17744}$$

### 4.2. Influence of the Blasting Method

**Figure 18:**
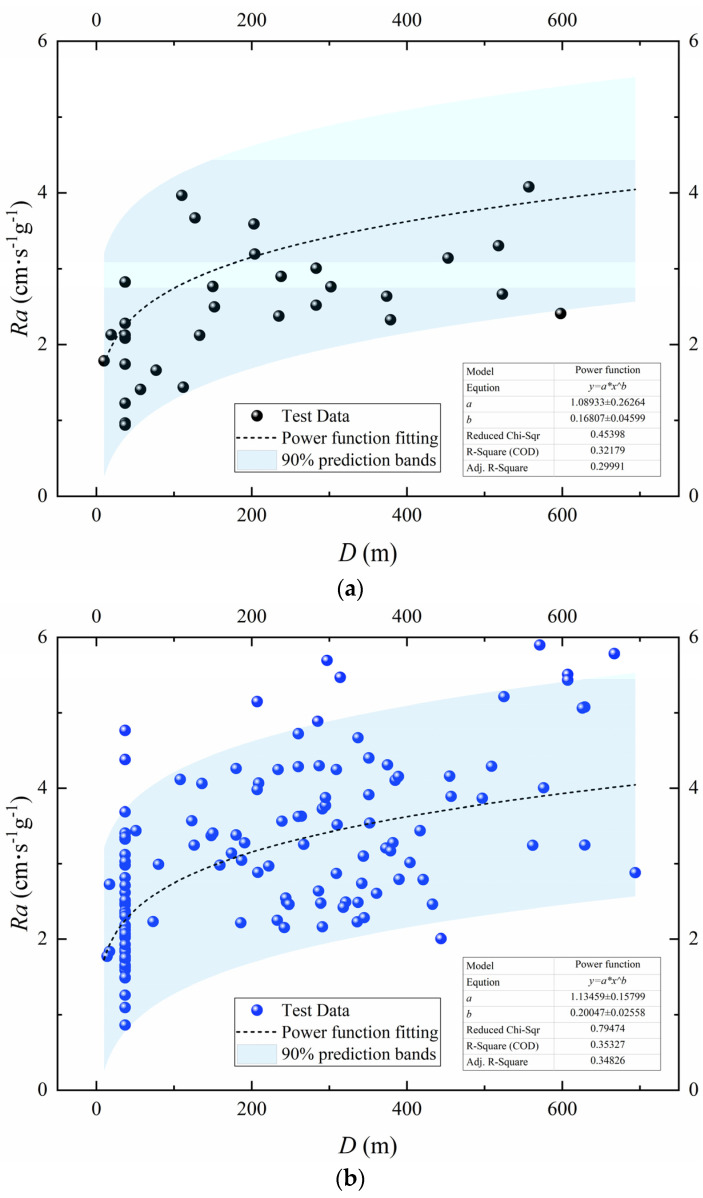
*Ra* of different blasting methods. (**a**) Pre-cracking blasting. (**b**) Bench blasting.

Two blasting methods, pre-splitting and bench blasting, were used in the blasting tests. Figure 18 illustrates the relationship between *Ra* and *D* for the two blasting methods with the following expressions:

(21) $$\mathrm{Pre}\text{-}\mathrm{splitting}\;\mathrm{blasting}\;Ra=1.08933D^{0.16807}$$

(22) $$\mathrm{Bench}\;\mathrm{blasting}\;Ra=1.13459D^{0.20047}$$

The *Ra* of pre-splitting blasting is significantly smaller than that of bench blasting. This result may be explained by the fact that the main purpose of pre-splitting blasting is to create fractures rather than fragmentation. During pre-splitting blasting, the charge explodes in a clamped state with only one free surface, which converts into a greater proportion of kinetic energy in the rock compared to bench blasting, and the vibration frequency is higher. Therefore, the *Ra* of pre-splitting blasting is less than that of bench blasting at the same propagation distance.

## 5. Velocity and Acceleration Attenuation Laws

Common attenuation equations for the blast vibration velocity, such as the Ambraseys–Hendron equation, the United States Bureau of Mines equation, etc., contain the same variables, but the specific forms are slightly different. Herein, the Ambraseys–Hendron equation is selected for analysis, and its expression is:

(23) $$\mathrm{PPV}=K{\left(Q^{1/3}/D\right)}^{\alpha }$$

where *Q* is the maximum charge per delay (kg), and *K* and α are the site constants. α represents the attenuation characteristics of the site, which is called the attenuation exponent. The larger the value, the greater the attenuation.

For comparative analysis, a similar expression of the attenuation law of acceleration is adopted:

(24) $$\mathrm{PPA}=K_{a}{\left(Q^{1/3}/D\right)}^{\alpha_{a}}$$

where $K_{a}$ and $\alpha_{a}$ are the site constants of acceleration.

**Figure 19:**
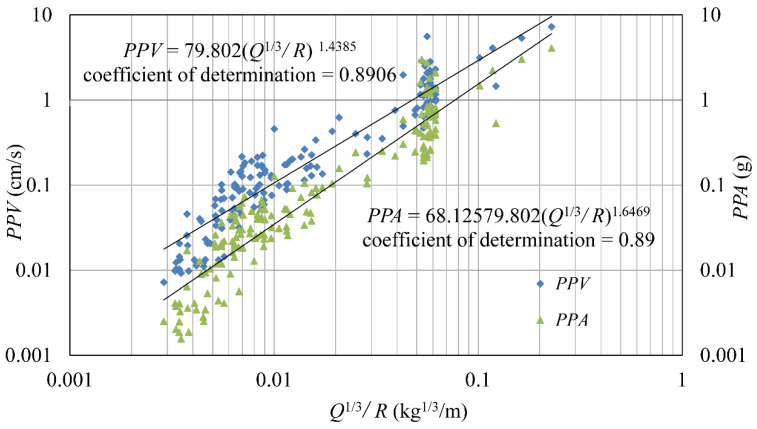
Attenuation law of PPV and PPA.

Figure 19 compares the attenuation law of the PPV and PPA, and the corresponding attenuation equations are expressed using Equations (25) and (26). It is apparent that the attenuation exponent of acceleration is greater than that of velocity, indicating that the attenuation of acceleration with distance is greater than that of velocity, resulting in a slow increase in Ra, further confirming the above analysis. In addition, Equation (25) is divided using Equation (26), and the Ra calculation equation including the propagation distance and the maximum charge per delay is also obtained, as shown in Formula (27). It can be seen that Ra has a positive correlation with the propagation distance and a negative correlation with the charge.

(25) $$\mathrm{PPV}=79.802{\left(Q^{1/3}/D\right)}^{1.4385}$$

(26) $$\mathrm{PPA}=68.125{\left(Q^{1/3}/D\right)}^{1.6469}$$

(27) $$Ra=1.171{\left(Q^{1/3}/D\right)}^{-0.2084}$$

## 6. Discussion

Generally speaking, WD can effectively characterize a larger number of signals. However, when the signal has substantial high-frequency information, WD fails to achieve a satisfactory decomposition and representation of high-frequency components. In contrast, WPD can execute an arbitrarily fine segmentation of the high-frequency part, thereby better characterizing such signals, making WPD superior to WD. Yet, when the SNR is small, WPD tends to match the high frequency (usually noise), leading to the potential removal of both the effective signal and noise, resulting in excessive noise reduction. For practicality and generality, the noise reduction evaluation index should be considered the standard, and the optimal noise reduction method should be determined according to comparison. This paper adopts this approach for noise reduction analysis.

Theoretically, the equivalent transformation between velocity and acceleration can be achieved using differentiation and integration. However, the calculated results reveal unavoidable errors. This is primarily because the transformation heavily depends on the authenticity and completeness of the original signals. Signals monitored using the seismometers contain various errors, including random errors and systematic errors. When the SNR is low, the denoised time–velocity/acceleration curve will inevitably mismatch with the original curve.

## 7. Conclusions

Velocity and acceleration are different manifestations of the same blast vibration, and they can refer to each other. Based on the analysis and discussion of the 164 sets of data monitored using two types of seismometers in the field blasting tests, the following primary conclusions can be drawn:

(1) For the transient, nonlinear, and wide-frequency characteristics of blast vibration signals, the combined CEEMDAN and WD/WDP method of noise reduction is presented. Based on the evaluation indexes (RMSE, SNR, and SSM), the optimal decomposition levels and wavelet basis functions suitable for different signals are determined.
(2) Vibration velocity and acceleration can be derived from each other according to differentiating and integrating. However, due to the inevitable noise in the monitored signals and the non-periodic, non-linear, and non-stationary characteristics of blast vibration signals, there is a certain discrepancy between the theoretical derivation and the measured signal. The results in this paper show that the relative error is concentrated between −25% and 30%. The removal of trend terms and noise reduction will greatly improve the accuracy of the transformation.
(3) There is a power function relationship between *Ra* and *D*, and Ra tends to be flat after increasing gradually. In addition, *Ra* shows a negative correlation with the maximum charge per delay. Due to the higher frequency of pre-splitting blasting, its *Ra* is lower than that of bench blasting at the same propagation distance.
(4) The attenuation exponent of acceleration is greater than that of velocity, indicating that the attenuation of acceleration, along with the propagation distance, is greater than that of velocity, resulting in a slow increase in *Ra.*

The current blasting-related specifications predominantly use velocity to evaluate blasting vibration. However, for special protection objects such as nuclear power stations and instruments controlled by inertial forces and considering that building design and seismic codes use acceleration loads for calculation and analysis, it is essential to improve the cross-reference between these two types of physical quantities. This study aims to provide a reference for these situations. Further research should be conducted to establish an Ra prediction equation involving more variables, such as topography and geology.

## Figures and Tables

**Table 1:** Statistics of blasting design parameters.

Total Charge Weight (kg)	Maximum Charge per Delay (kg)	Blast Hole Diameter (mm)	Charge Diameter (mm)	Row Spacing (m)	Hole Spacing (m)	Hole Depth (m)	Stemming Length (m)
48–864	4–12	76/90	32/70	1.8–2.5	1–3	1.3–12.77	1.0–3.9

**Table 2:** Relative error of integral and differential calculations.

Distance (m)	Relative Error Translated to Velocity (%)		Relative Error Translated to Acceleration (%)	
	Trend Item Not Removed	Remove Trend Item	Trend Item Not Removed	Remove Trend Item
10	7.75	1.67	−16.03	−16.04
235	52.02	28.13	−9.64	−9.63

## Data Availability

The corresponding authors can provide more details regarding this research through email.
